# Treatment of Arteriosclerosis

**Published:** 1907-03

**Authors:** 


### ABSTRACTS.

### Treatment of Arteriosclerosis.

LUDWIG WEIL, Stuttgart, has made extensive clinico-therapeutic studies of arteriosclerosis (*Medicin. Correspondenz-Blatt d. Wuerttemb, aerztl, Landesverein.* Sept. 15, 1906). He concludes that the calcium-free diet as also the administration of acids, in order to decalcify the vessels, is useless and injurious. His experimentation shows that in arteriosclerosis the blood and the tissues are impoverished of salts as has been asserted by Trunecek. This is all the more striking because the blood is otherwise entirely normal. The nitrogen retention is not increased, so that the excretory power of the organism seems unimpaired and the solid contents of the blood and with it probably the viscosity are quite high, while specific gravity appears normal.

The arteriosclerotic excretes too much chlorine and there is often a considerable diminution in lime and magnesium, some diminution in the phosphates, and a small increase in ammonia and acidity. The blood contains little chlorides, the urine a great deal.

The best treatment consists in the administration of a mixture of salts corresponding as nearly as possible to the salt of the blood. From this mixture the body abstracts what it can use. Such a mixture he considers antisclerosin, with which his results were indubitably good. Iodides, diuretin, and similar preparations, are of course not rendered superfluous by its use. A sudden overwhelming of the body with the salts is only rarely of benefit; there often follows catharsis and general indisposition. He therefore prefers the gradual insinuation of the medicament. His procedure is to give an antisclerosin tablet twice daily for three days, then thrice daily for a further three days, and then in corresponding intervals increases the daily quantity to four, five and six tablets.

Dr. O. Burwinkel, Nauheim and San Remo, (*Deutsche Aerzte-Zeitung,* Aug. 15, 1905), writes on the pathogenesis and therapy of arteriosclerosis. He refers to Trunecek's serum, which consists of the inorganic blood salts and which Tessier, Levy and Goldschmidt used with success. As the subcutaneous injection is exceedingly painful, he uses the antisclerosin tablets made by Natterer. He believes that in some cases he has seen benefit from it, especially with regard to the severity of the anginous difficulties.

